# Homocysteine Thiolactone Detoxifying Enzymes and Alzheimer’s Disease

**DOI:** 10.3390/ijms25158095

**Published:** 2024-07-25

**Authors:** Hieronim Jakubowski

**Affiliations:** 1 Department of Biochemistry and Biotechnology, University of Life Sciences, 60-637 Poznań, Poland; jakubows@rutgers.edu; Tel.: +48-973-972-8733; Fax: +48-973-972-8981; 2 Department of Microbiology, Biochemistry and Molecular Genetics, New Jersey Medical School, Rutgers University, International Center for Public Health, Newark, NJ 07103, USA

**Keywords:** BLMH, BPHL, PON1, homocysteine thiolactone, PHF8, mTOR signaling, autophagy, Alzheimer’s disease

## Abstract

Elevated levels of homocysteine (Hcy) and related metabolites are associated with Alzheimer’s disease (AD). Severe hyperhomocysteinemia causes neurological deficits and worsens behavioral and biochemical traits associated with AD. Although Hcy is precluded from entering the Genetic Code by proofreading mechanisms of aminoacyl-tRNA synthetases, and thus is a non-protein amino acid, it can be attached to proteins via an *N*-homocysteinylation reaction mediated by Hcy-thiolactone. Because *N*-homocysteinylation is detrimental to a protein’s function and biological integrity, Hcy-thiolactone-detoxifying enzymes—PON1, BLMH, BPHL—have evolved. This narrative review provides an account of the biological function of these enzymes and of the consequences of their impairments, leading to the phenotype characteristic of AD. Overall, accumulating evidence discussed in this review supports a hypothesis that Hcy-thiolactone contributes to neurodegeneration associated with a dysregulated Hcy metabolism.

## 1. Introduction

Alzheimer’s disease (AD), the most common cause of dementia, is a major health problem in aging populations [1]. AD is characterized by the extracellular accumulation of amyloid β (Aβ) and the intracellular accumulation of neurofibrillary tangles of the hyperphosphorylated tau protein, leading to neuronal death. Mutations in the amyloid precursor protein (APP), presenilin 1 (PSEN1), and presenilin 2 (PSEN2) are responsible for the familial early-onset AD, which is relatively rare [2]. Lifestyle and environmental factors have emerged as modulators of susceptibility to AD [3,4]. For example, an insult to the brain, such as exposure to pesticides or metals, is an environmental risk factor for AD. Lifestyle factors, such as nutrition, exercise, and level of education, are thought to play a protective role, delaying the onset and/or severity of the disease [5]. However, the causes of the most common sporadic late-onset AD are largely unknown, and no effective therapy is available [6]. Thus, the identification of novel risk factors and their mechanisms of action has important public health implications. Hyperhomocysteinemia (HHcy) is an emerging risk factor for AD [7,8,9,10]. However, the mechanisms underlying the involvement of HHcy in AD are not fully understood. Specifically, it is not clear whether elevated levels of homocysteine (Hcy) itself or its downstream metabolites, such as Hcy-thiolactone (HTL) and *N*-homocysteinylated proteins, can be involved in AD.

Cystathionine β-synthase (CBS) deficiency, the most prevalent inborn error in the sulfur amino acid metabolism in humans [11,12,13], is biochemically characterized by severe HHcy, i.e., severely elevated levels of Hcy [14] and its metabolites, Hcy-thiolactone and *N*-Hcy-protein [15,16,17,18]. CBS deficiency affects the central nervous system and causes severe learning and intellectual disability [11,19], reduced IQ [20], psychosis, obsessive–compulsive disorder, and behavior/personality disorders [21]. Accelerated brain atrophy associated with HHcy has been reported in healthy elderly individuals [22,23], alcoholic patients [24], and AD patients [25], who also show upregulated brain mTOR signaling [26,27]. These phenotypes are also seen in an animal model of human CBS deficiency, the *Cbs*^−/−^ mouse. Specifically, in the *Cbs*^−/−^ mouse model, severe HHcy is accompanied by neurological impairments and cognitive deficiency characterized by attenuated problem-solving abilities, learning, and short- and long-term memory [28,29].

Studies in *Cbs*^−/−^ mice and mouse neuroblastoma cells revealed that Hcy and its metabolites influenced molecular mechanisms underlying these neurological impairments [30]. Specifically, the expression of the histone demethylase Phf8 was reduced, while the methylated histone H4K20me1, mTOR signaling, and App were increased in brains of *Cbs*^−/−^ mice compared with *Cbs*^+/−^ sibling controls. Autophagy-related proteins Becn1, Atg5, and Atg7 were downregulated, while neurodegeneration-related neurofilament-L (Nfl) and glial fibrillary acidic protein (Gfap) were upregulated in *Cbs*^−/−^ brains. Treatments with Hcy-thiolactone, *N*-Hcy-protein, or Hcy (which were all severely elevated in *Cbs*^−/−^ mice; [15,16,17,18]) or *Cbs* gene silencing by RNA interference significantly reduced Phf8 expression and increased total H4K20me1 as well as mTOR promoter-bound H4K20me1 in mouse neuroblastoma N2a and N2a-APPswe cells. This caused transcriptional mTOR upregulation, autophagy downregulation, and significantly elevated APP and Aβ levels. The *Phf8* gene silencing increased Aβ, but not APP, levels. These findings show that Phf8 regulates Aβ synthesis and suggest that neuropathy seen in mouse Cbs deficiency is mediated by Hcy metabolites, which transcriptionally dysregulate the Phf8 → H4K20me1 → mTOR → autophagy pathway, thus increasing Aβ accumulation [30]. As will be discussed below in Section 5, similar molecular changes were observed in mouse models of AD that were impaired in their abilities to detoxify Hcy-thiolactone.

Because *N*-homocysteinylation by Hcy-thiolactone is detrimental to a protein’s function and biological integrity [17,18,31,32], enzymes detoxifying Hcy-thiolactone have evolved: serum paraoxonase 1 (PON1) [33], cytoplasmic bleomycin hydrolase (BLMH) [34], and mitochondrial biphenyl hydrolase-like (BPHL) enzyme [35,36,37], all of which hydrolyze Hcy-thiolactone to Hcy. The enzymatic detoxification reaction protects proteins from *N*-homocysteinylation [33,38] because it eliminates Hcy-thiolactone, which would otherwise damage them [17,18,31].

Accumulating evidence suggests that the Hcy-thiolactone-hydrolyzing enzymes PON1, BLMH, and BPHL play an important role in the central nervous system. This review provides an overview of the current understanding of the biological function of Hcy-thiolactone-detoxifying enzymes and of the consequences of their impairment, leading to the phenotype characteristic of AD. To provide a context for the discussion of Hcy-thiolactone-detoxifying enzymes, Hcy metabolism, biogenesis, and the chemical biology of Hcy-thiolactone and *N*-homocysteinylated proteins are also briefly summarized. Taken together, the findings discussed in this review support the hypothesis that Hcy-thiolactone contributes to neurodegeneration associated with a dysregulated Hcy metabolism.

## 2. Homocysteine Metabolism

**Figure 1:**
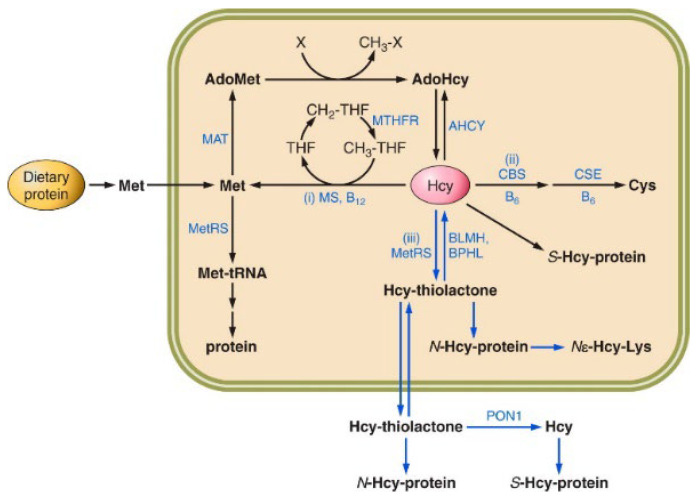
Schematic representation of homocysteine metabolism in humans and mice: the remethylation (i), transsulfuration (ii), and homocysteine (Hcy)-thiolactone (iii) pathways. Protein-metabolism-related reactions involving Hcy are highlighted by blue arrows. The rectangle symbolizes the cell, the outside area is plasma, and the oval labelled “Dietary protein” represents the digestive tract. See text for description. AdoMet, adenosylmethionine; BPHL, biphenyl hydrolase-like; CBS, cystathionine β-synthase; MAT, Met S-adenosyltransferase; Met, methionine; MetRS, methionyl-tRNA synthetase; MS, Met synthase; MTHFR, methylenetetrahydrofolate reductase; and THF, tetrahydrofolate. Reproduced with permission from Jakubowski [17].

Hcy was synthesized in 1935 by the reduction [39] in disulfide homocysteine (Hcy-S-S-Hcy), obtained in 1932 by boiling Met in sulfuric acid [40]. The article describing the first synthesis of Hcy also reported the synthesis of Hcy-thiolactone from Hcy in strongly acidic solutions [39]. Later studies clarified the role of Hcy formed as a product of Met metabolism in a reaction catalyzed by the enzyme AHCY [41] (Figure 1) and as a precursor of the sulfur amino acids Met (reaction (i)) and cysteine (reaction (ii)) [42] and of the thioester Hcy-thiolactone (reaction (iii)) [17,18] (Figure 1).

In humans and other mammals, Hcy is generated from Met as a byproduct of S-adenosylmethionine (AdoMet)-mediated methylation reactions [42]. Met, an essential amino acid supplied with protein in a diet, is released in the digestive system, taken up by epithelium, and metabolized to Hcy via the Met → AdoMet → AdoHcy → Hcy pathway in various organs (Figure 1). Hcy is then metabolized to Hcy-thiolactone by methionyl-tRNA synthetase (MetRS or MARS) [32], remethylated back to Met, or transsulfurated to Cys [42] (Figure 1). The genetic or dietary deficiencies affecting transsulfuration (CBS, CSE) or remethylation (MS, MTHFR) enzymes (Figure 1) lead to the accumulation of Hcy, Hcy-thiolactone [15,32], and *N*-Hcy-protein [16,32], and are associated with various pathologies in humans [8,17,18].

## 3. Homocysteine Thiolactone

Hcy-thiolactone, an intramolecular thioester of Hcy, was first synthesized in 1934 by boiling methionine (Met) with hydriodic acid from [43]. A more recent study showed that the digestion of L-Met with hydriodic acid yields a D,L-Hcy-thiolactone racemate [44]. Because the recovery of Hcy-thiolactone was quantitative [43], the hydriodic acid digestion provided a convenient method for the preparation of D,L-[^35^S]Hcy-thiolactone [44], which facilitated the elucidation of Hcy-thiolactone metabolic pathways [17,18,31,32,45].

The enzymatic conversion of Hcy to Hcy thiolactone in an editing reaction of MARS prevents the access of Hcy to the Genetic Code [46,47] and is universal, occurring in all cell types and organisms investigated so far, from bacteria to humans [17]. The Hcy editing reaction is the only known mechanism of Hcy-thiolactone biosynthesis [46] (Figure 1). The fundamental role of MARS in Hcy-thiolactone biosynthesis in mammalian cells has been established by showing that Chinese hamster ovary cells harboring a temperature-sensitive mutation in the gene encoding MARS are unable to synthesize Hcy-thiolactone at a non-permissive temperature [17] and that Hcy-thiolactone formation in human endothelial cells was inhibited by Met [32].

**Figure 2:**
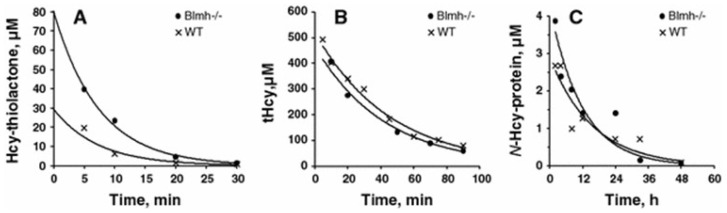
Kinetics of plasma Hcy-thiolactone (**A**), total Hcy (**B**), and *N*-Hcy-protein (**C**) turnover in mice. For Hcy-thiolactone (**A**) and total Hcy (**B**) turnover experiments, mice were injected i.p. with 600 nmol L-Hcy-thiolactone/g body weight. For *N*-Hcy-protein (**C**) turnover experiments, 2,850 nmol L-Hcy-thiolactone/g body weight was used. Metabolites were analyzed at indicated times after injection, and data points were fitted to an exponential equation [A*^t^*] = [A^0^]·e^−*k·t*^, where *k* is a first-order rate constant, [A*^t^*] is a metabolite concentration measured at time *t*, and [A^0^] is a metabolite concentration extrapolated to time zero. Representative kinetics obtained for individual knockout *Blmh*^−/−^ (•) and wild-type *Blmh*^+/+^ (x) mice are shown. Reproduced with permission from Borowczyk et al. [48].

In mice, Hcy-thiolactone is quickly cleared from the body (t_1/2_ = 5.1 min) [48,49], about six times faster than Hcy (t_1/2_ = 31.8 min) [48,49] and 120-times faster than *N*-Hcy-protein (t_1/2_ = 10.2 h) [48] (Figure 2). Efficient Hcy-thiolactone clearance is responsible for its relatively low levels, compared with Hcy and *N*-Hcy-protein levels in humans and mice [17].

## 4. *N*-Homocysteinylated Proteins

Hcy-thiolactone is harmful because of its ability to chemically modify protein lysine residues, which impairs the protein structure and function, as first shown for human *N*-homocysteinylated (*N*-Hcy)-albumin [50], whose K525Hcy modification increased the protein’s susceptibility to oxidation and proteolysis [50]. Two other *N*-homocysteinylated lysine residues were identified in human albumin in vivo: K137Hcy and K212Hcy; of these, K212Hcy was more abundant in male than female mice [17]. Notably, albumin, a classical globular protein with predominantly α-helical secondary structures, was converted by *N*-homocysteinylation to amyloid-like aggregates with prevailing β-sheet secondary structures [51].

Subsequent studies showed that the *N*-homocysteinylation of other proteins conferred on them immunogenic [17], atherogenic [17], thrombogenic [52], amyloidogenic [51], neuropathic [53,54,55,56], and oncogenic [57] properties.

In cell cultures, *N*-Hcy-protein biogenesis positively correlated with the concentrations of its precursors Hcy and Hcy-thiolactone and with the MARS activity [32]. Vitamin B_12_ and folate, cofactors of Hcy-metabolizing enzymes, inhibited *N*-Hcy-protein biogenesis [58]. Methionine, which inhibits the MARS-dependent metabolic conversion of Hcy to Hcy-thiolactone, also inhibited *N*-Hcy-protein biogenesis [32]. The antifolate drug aminopterin, which prevents metabolic conversion of Hcy to Met, increased *N*-Hcy-protein biogenesis [45]. In humans, *N*-Hcy-protein biogenesis increased in *CBS* and *MTHFR* deficiencies [16] and was influenced by *PON1* polymorphism [38] and PON1 arylesterase activity [59]. In mice, *N*-Hcy-protein biogenesis is affected by the diet and *Cbs*, *Mthfr*, *Pcft*, *Pon1*, and *Blmh* genotypes [17].

Additional evidence supporting the mechanism of *N*-Hcy-protein biogenesis comes from the identification by mass spectrometry of specific *N*-Hcy-lysine (KHcy) residues in proteins: K525Hcy, K212Hcy, and K137Hcy in human and mouse serum albumin [17]; αK562Hcy, βK344Hcy, and γK385Hcy in human fibrinogen [17]; K160Hcy in mouse collagen [17]; 5 KHcy residues (K14Hcy, K18Hcy, K23Hcy, K27Hcy, and K56Hcy) in histone H3 from HTL-treated HEK293 T cells [60]; 5 KHcy residues (K32Hcy, K121Hcy, K338Hcy, K1173, and K1812) in ATR from HCT116 cells [57]; K1218Hcy in dynein from rat brain [53]; 304 KHcy residues in proteins from HTL-treated HeLa cells [61]; 2,525 KHcy residues in 870 different proteins from NE4C cell [56]; H3K79Hcy and other histone KHcy residues in human fetal NTD brain [62]; K411Hcy in MAP1 from rat brain [63]; K80Hcy in α-synuclein from mouse brain [54]; and K182Hcy in DJ-1 from HEK293 cells [55].

## 5. Hcy-Thiolactone Hydrolyzing Enzymes

### 5.1. Paraoxonase 1

Paraoxonase 1 (PON1), named for its ability to hydrolyze the organophosphate pesticide paraoxon [64,65], is the first enzyme that was found to use Hcy-thiolactone as a physiological substrate [33] (Table 1). PON1, a monomeric enzyme of 43 kDa molecular weight, synthesized in the liver and carried in the blood attached to a minor subclass of high-density lipoprotein (HDL) that represents 5% of total HDL [66], is present in many organs, including the brain [67]. It protects from organophosphate toxicity in agricultural workers [68]. PON1 arylesterase and paraoxonase activities and PON1-192R genotype have been reported to protect from major adverse cardiovascular events in patients with coronary artery disease [69,70] and chronic kidney disease [71]. Low PON1 Hcy-thiolactone hydrolytic activity predicted worse long-term mortality in [72]. In a general population, PON1 arylesterase activity predicted major adverse cardiovascular events [73]. However, the EPIC-Norfolk prospective population study found that the PON1 genotype and activity did not predict the risk of future coronary artery disease [74].

In mice, Pon1 protects from atherosclerosis induced by a high-fat diet [75] or ApoE depletion [76]. Pon1 also protects mice from renal lipotoxicity by reducing the expression of oxidative stress/inflammation-related genes and inhibiting mTOR expression [77]. Cardio protection by PON1 can be due to its apparent antioxidative function mediated by interactions of PON1 with redox-response-related proteins [78,79] and its ability to detoxify Hcy-thiolactone [33,49,80], which attenuate lipid/protein peroxidation [69,75,81] and protein *N*-homocysteinylation [38,49,59].

PON1 has also been implicated in AD [82,83,84,85], which may not be unexpected given that AD has a significant vascular component [86]. Meta-analysis of the data published up to February 2023 (seven studies involving a total of 615 subjects, 281 AD patients, and 356 controls) showed that PON1 arylesterase activity, reflecting levels of the PON1 protein [87,88], was significantly reduced in AD patients compared with controls [89]. Exposure to organophosphates is associated with lower PON1 activity [85] and has been linked to neurological disorders including AD, Parkinson’s disease (PD), intellectual disability, attention deficit hyperactivity disorder (ADHD), autism, and other developmental neuropathies [90]. Pesticide exposure in AD patients was associated with significantly lower PON1 arylesterase activity, which was accompanied by lower activities of the antioxidant enzymes SOD and GPX [85]. PON1 arylesterase activity was significantly reduced in AD and dementia patients compared with healthy controls [91,92,93,94] and negatively correlated with the extent of AD-related cognitive decline [95]. In mild cognitive impairment patients, PON1 arylesterase activity predicted global cognition, verbal episodic memory, and attention/processing speed [96]. *ApoE*^−/−^*Pon1*^−/−^ mice with severe carotid atherosclerosis [76] also showed markers of AD and impaired brain vasculature at 14 months, although it was not clear whether brain pathology occurred due to *ApoE*^−/−^, *Pon1*^−/−^, or both knockouts [97]. In a Tg2576 mouse AD model, immunohistochemical signals for Pon1 surrounded Aβ plaques in various brain regions but could not be assigned to any cell type [98].

#### 5.1.1. Consequences of Pon1 Gene Ablation

**Figure 3:**
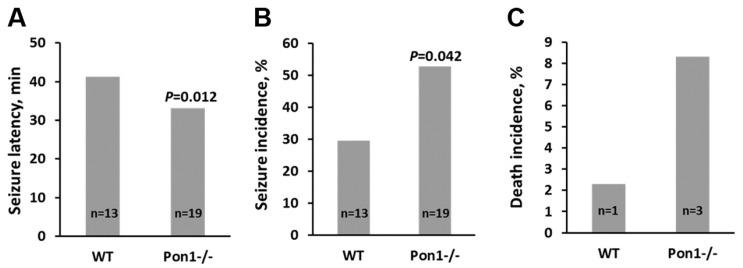
Latenecy (**A**) and incidence (**B**) of *L*-Hcy-thiolactone-induced seizures and death incidence (**C**) in *Pon1*^−/−^ mice relative to their *Pon1*^+/+^ siblings (WT). *L*-Hcy-thiolactone was injected i.p. into *Pon1*^−/−^ (n = 19) and *Pon1*^+/+^ (WT, n = 13) mice (3.7 μmol/g body weight); the animals were monitored for 90 min. Data from Borowczyk et al. [49].

*Pon1* gene deletion in mice diminished their Hcy-thiolactone hydrolyzing ability (Table 2), causing Hcy-thiolactone accumulation in the brain, kidney, and urine. *Pon1*^−/−^ mice exhibited significantly increased neurotoxic response to Hcy-thiolactone injections compared with their *Pon1*^+/+^ siblings [49] (Figure 3). *Pon1*^−/−^ mice were also more susceptible to organophosphates and other neurotoxic agents [68,75] and to atherosclerosis [75,76].

Studies of brain proteomes in *Pon1*^−/−^ vs. *Pon1*^+/+^ mice showed that Pon1 interacts with diverse cellular processes, such as energy metabolism, anti-oxidative defenses, cell cycle, cytoskeleton dynamics, and synaptic plasticity, that are essential for brain homeostasis. The findings that Pon1 depletion influenced the expression of oxidative-stress-responsive proteins associated with AD, such as Sod1, Prdx2, and DJ-1 [17], suggest that Pon1 involvement in oxidative stress is indirect.

Clusterin (CLU, APOJ), involved in the transport of amyloid beta (Aβ) from the plasma to the brain in humans (reviewed in [2]), is carried on a distinct HDL subspecies that contains three major proteins: PON1, CLU, and APOA1 [66]. Notably, levels of Clu (ApoJ) were significantly elevated in the plasma of *Pon1*^−/−^ vs. *Pon1*^+/+^ mice [78]. Taken together, these findings suggest that PON1 plays an important role in the CNS.

#### 5.1.2. Pon1 Depletion Downregulates Phf8, Upregulates mTOR Signaling, and Inhibits Autophagy

That PON1 plays an important role in the CNS is further supported by a recent study using a new mouse model of AD, the *Pon1*^−/−^5xFAD mouse, which elucidated molecular mechanism by which Pon1 maintains CNS homeostasis and protects the brain from the accumulation of Aβ, a hallmark of AD [99]. The study showed that Pon1 depletion, which causes the accumulation of Hcy-thiolactone and the *N*-Hcy-protein in mice [49], downregulated the histone demethylase Phf8 and upregulated the H4K20me1 epigenetic mark in brains of *Pon1*^−/−^ mice and in Pon1-silenced mouse neuroblastoma N2a-APPswe cells [99]. The depletion of Pon1 increased H4K20me1 binding to the mTOR promoter, demonstrated in mouse neuroblastoma N2a-APPswe cells, and upregulated mTOR signaling, which in turn inhibited the autophagy flux.

#### 5.1.3. Pon1 Depletion Upregulates App and Aβ

In mouse neuroblastoma N2a-APPswe cells and in brains of *Pon1*^−/−^5xFAD mice, Pon1 depletion upregulated the amyloid precursor protein (App) and amyloid beta (Aβ) [99]. Treatments with *N*-Hcy-protein and Hcy-thiolactone induced similar biochemical changes in App and Aβ levels in the mouse neuroblastoma cells.

These findings provide direct mechanistic evidence linking Hcy-thiolactone and *N*-Hcy-protein with dysregulated mTOR signaling and its downstream consequences, such as downregulation of autophagy and upregulation of Aβ. This mechanism is further supported by findings showing that Phf8 depletion by RNA interference affected mTOR, autophagy, APP, and Aβ, as did Pon1 depletion or treatments with Hcy-thiolactone or *N*-Hcy-protein. These findings also suggest that Pon1 is a negative regulator of mTOR signaling by controlling levels of Hcy metabolites that affect the binding of H4K20me1 at the mTOR promotor and define a neuroprotective mechanism by which Pon1 protects from amyloidogenic App processing to Aβ in the mouse brain [99].

#### 5.1.4. Pon1 Interacts with App but Phf8 Does Not

Although the depletion of Pon1 downregulated Phf8 and upregulated APP in the brains of *Pon1*^−/−^5xFAD mice, Phf8 depletion did not change the APP level [99], suggesting that Pon1 interacts with APP in the mouse brain while Phf8 does not. The nature of the Pon1-APP interaction, whether it is direct or indirect, remains to be elucidated.

**Figure 4:**
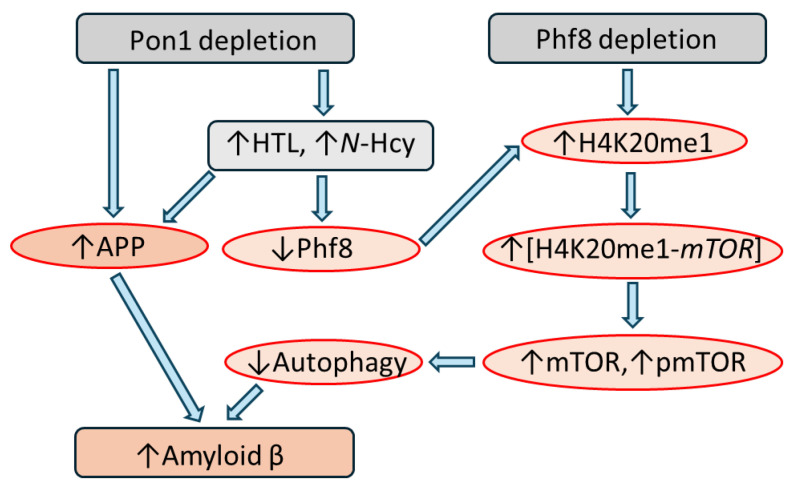
Hypothetical pathways leading to Aβ generation in *Pon1*^−/−^5xFAD mice. Up and down arrows show the direction of changes. Blmh, bleomycin hydrolase; Hcy, homocysteine; HTL, Hcy-thiolactone; APP, amyloid beta precursor protein; mTOR, mammalian target of rapamycin; pmTOR, phospho-mTOR; Phf8, plant homeodomain finger protein 8. H4K20me1-*mTOR* represents H4K20me1 bound at the *mTOR* promoter.

Pon1 depletion downregulated Phf8 and upregulated Aβ in the brains of *Pon1*^−/−^5xFAD mice and in mouse neuroblastoma N2a-APPswe cells. In contrast, Phf8 depletion upregulated Aβ, although it did not affect the APP level [99]. This suggests that two pathways are involved in Aβ generation in a Pon1-depleted mouse brain and neural cells. One pathway involves Hcy-thiolactone and *N*-Hcy-protein metabolites, which upregulate APP, while another pathway, mediated by Phf8, H4K20me1, and mTOR, involves impaired Aβ clearance due to downregulated autophagy (Figure 4).

#### 5.1.5. Similar Effects of Pon1 Depletion and Hcy-Thiolactone/N-Hcy-Protein on Pathways Leading to Aβ

Interestingly, Pon1 depletion induced changes in the Phf8 → H4K20me1 → mTOR → autophagy pathway in a *Pon1*^−/−^5xFAD mouse brain and in Pon1-silenced neuroblastoma cells that mimicked the changes induced by HHcy in a *Pon1*^+/−^5xFAD mouse brain and in Hcy-thiolactone- or *N*-Hcy-protein-treated mouse neuroblastoma N2a-APPswe cells [99]. Pon1 depletion or HHcy similarly increased the accumulation of Aβ in the brain. An earlier work showed that biochemical outcomes of Pon1 depletion and HHcy were identical: HHcy elevated Hcy-thiolactone and *N*-Hcy-protein [17,18], as did Pon1 depletion [49,59]. Pon1 depletion by RNA interference or treatments with Hcy-thiolactone or *N*-Hcy-protein similarly elevated Aβ in mouse neuroblastoma cells. Taken together, these findings suggest that increased accumulation of Aβ in a Pon1-depleted brain is mediated by the effects of Hcy metabolites on mTOR signaling and autophagy. These findings also suggest that Pon1 is a negative regulator of mTOR signaling by controlling Hcy-related metabolite levels that influence the extent of H4K20me1 binding at the mTOR promoter.

### 5.2. Bleomycin Hydrolase

Human BLMH [100,101], named for its ability to hydrolyze the anticancer drug bleomycin, is the second enzyme that was found to use Hcy-thiolactone as a physiological substrate [34] (Table 2). BLMH, a cytoplasmic enzyme expressed in various organs, has a quaternary structure like the 20 S proteasome and is a member of the self-compartmentalizing cysteine proteases family [102]. In addition to being studied in relation to Hcy toxicity [34,103] and Alzheimer’s disease [104,105,106], BLMH was also studied in the field of protein turnover [107,108], cancer therapy [101,109,110], keratinization disorders [111], and asthma [112]. The I443V polymorphic site located in the C-terminal domain important for the activity of the human BLMH is associated with a risk of AD in some [105,113] but not all studies [114,115,116].

A cytoplasmic Hcy-thiolactone-hydrolyzing activity was originally purified from human placenta and identified by proteomic and biochemical analyses as BLMH [34]. Substrate specificity studies showed that the human BLMH exhibits absolute stereo-specificity for *L*-Hcy-thiolactone, the preferred natural substrate, and does not hydrolyze *D*-Hcy-thiolactone (Table 1). Methyl esters of sulfur-containing amino acids such as *L*-Cys and *L*-Met were also hydrolyzed, while *D*-Met methyl ester was not. *L*-homoserine lactone, γ-thiobutyrolactone, and other *L*-amino acids were not hydrolyzed by the human BLMH [34].

The recombinant human and yeast BLMH, expressed in *E. coli*, exhibit Hcy-thiolactone-hydrolyzing activity like that of the corresponding native enzymes. Active site mutation C73A in the human BLMH and H369A in the yeast BLMH inactivate their Hcy-thiolactone hydrolyzing activity [34].

#### 5.2.1. Consequences of Blmh Gene Ablation

**Figure 5:**
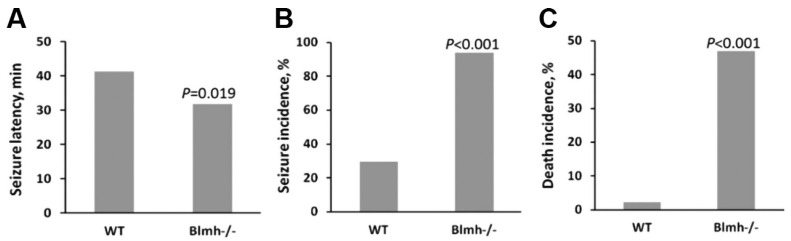
Latenecy (**A**) and incidence (**B**) of *L*-Hcy-thiolactone-induced seizures and death incidence (**C**) in *Blmh1*^−/−^ mice relative to their *Blmh1*^+/+^ littermates (WT). *L*-Hcy-thiolactone was injected i.p. into *Blmh1*^−/−^ (n = 32) and *Blmh1*^+/+^ (WT, n = 44) mice (3.7 μmol/g body weight); the animals were monitored for 90 min. Data from Borowczyk et al. [48].

In mice, the deletion of the *Blmh* gene [117] diminished the animal’s ability to detoxify Hcy-thiolactone, which led to its accumulation in the brain, kidney, and urine [48], and resulted in several brain-related phenotypes, such as astrogliosis and behavioral changes [118], and increased neurotoxic response to Hcy-thiolactone injections [48] (Figure 5), in addition to skin-related (tail dermatitis [117]) and immune-response-related phenotypes (impaired antigen presentation [119]).

The neurotoxic response induced by Hcy-thiolactone was more severe in *Blmh1*^−/−^ mice (90% seizure incidence and 48% death incidence, Figure 5) than in *Pon1*^−/−^ mice (52% seizure incidence and 8% death incidence, Figure 3). Hcy-thiolactone has been shown to inhibit Na^+^/K^+^-ATPase activity in the rat brain cortex, hippocampus, and stem, which may account at least in part its neurotoxic properties [120]. Interestingly, the inhibition of the neuronal nitric oxide synthase with 7-nitroindazole increased the severity of seizures induced by Hcy-thiolactone in a rat model [121].

In the brains of AD patients, the Hcy-thiolactonase and aminopeptidase activities of BLMH were significantly decreased compared with control brains, suggesting that the attenuated BLMH activity contributes to the pathology of AD [103]. The serum BLMH level was significantly reduced in Parkinson’s disease (PD) patients who responded to the therapeutic deep brain stimulation [122], a treatment recommended for advanced stages of PD [123]. The levels of BLMH in extracellular vesicles from the cerebrospinal fluid were significantly lower in amyotrophic lateral sclerosis patients compared with healthy controls [124]. Proteomic studies of a *Blmh*^−/−^ mouse brain showed that Blmh affects various cellular processes, which are important for brain homeostasis, including synaptic plasticity, cytoskeleton dynamics, cell cycle, energy metabolism, and antioxidant defenses [17]. Taken together, these findings suggest that Blmh plays an important role in the CNS.

#### 5.2.2. Blmh Depletion Downregulates Phf8, Upregulates mTOR Signaling, and Inhibits Autophagy

To elucidate the molecular mechanism by which Blmh maintains CNS homeostasis and protects the brain from the accumulation of Aβ, a hallmark of AD, a recent study examined biochemical and behavioral traits related to AD in a new mouse model, the *Blmh1*^−/−^5xFAD mouse [125]. 5xFAD mice overexpress the K670N/M671L (Swedish), I716V (Florida), and V717I (London) mutations in human APP (695) and the M146L and L286V mutations in human PS1 associated with familial early-onset AD and accumulate elevated levels of Aβ42 beginning around 2 months of age [126].

The study showed that Blmh depletion, which causes the accumulation of Hcy-thiolactone and *N*-Hcy-protein in mice [48], downregulated the histone demethylase Phf8 and upregulated the H4K20me1 epigenetic mark in the brains of *Blmh*^−/−^ and *Blmh*^−/−^5xFAD mice [125]. These findings were recapitulated in Blmh-silenced mouse neuroblastoma N2a-APPswe cells that harbor a human APP transgene with the K670N and M671L Swedish mutations associated with familial early-onset AD [127]. Blmh depletion increased H4K20me1 binding to the mTOR promoter (demonstrated in N2a-APPswe cells) and upregulated mTOR signaling, which in turn inhibited the autophagy flux in N2a-APPswe cells and in the brains of *Blmh*^−/−^5xFAD mice.

#### 5.2.3. Blmh Depletion Upregulates App and Aβ and Worsens Cognitive and Neuromotor Deficits

Blmh depletion upregulated App and Aβ in mouse neuroblastoma cells and in *Blmh*^−/−^5xFAD mouse brains [125]. Treatments with *N*-Hcy-protein and Hcy-thiolactone induced similar biochemical changes in mouse neuroblastoma cells. These biochemical changes were associated with cognitive and neuromotor deficits in *Blmh*^−/−^ and *Blmh*^-/-^5xFAD mice. For example, one-year-old *Blmh*^−/−^5xFAD mice scored worse compared with *Blmh*^+/+^5xFAD animals in the ovel object recognition test, indicating impaired memory, and in the hindlimb and cylinder tests, indicating sensorimotor impairments. Four-month-old *Blmh*^−/−^ mice, which did not accumulate Aβ, showed similar memory and sensorimotor impairments compared with *Blmh*^+/+^ animals. These findings show that the absence of the Blmh protein causes memory and sensorimotor impairments independently of the Aβ-producing transgene [48].

Neurological impairments seen in *Blmh*^−/−^ and *Blmh*^−/−^5xFAD mice are likely to be caused, at least partly, by Phf8 depletion, which does occur in *Blmh*^−/−^ brains [125]. That Phf8 depletion could account for the neurological deficits in *Blmh*^−/−^ and *Blmh*^−/−^5xFAD mice is supported by findings showing that PHF8 depletion in humans causes neurological impairments such as intellectual disability, autism spectrum disorder, and attention deficit hyperactivity disorder [128,129] and that *Phf8*^−/−^ mice also show similar neuropathies [130].

#### 5.2.4. Treatments with Hcy-Thiolactone or N-Hcy-Protein Mimicked the Effects of Blmh Depletion

Notably, treatments with Hcy-thiolactone or *N*-Hcy-protein mimicked the effects of Blmh depletion by siRNA treatments in the mouse neuroblastoma cells [125]. For example, Hcy-thiolactone, *N*-Hcy-protein, or Blmh depletion inhibited Phf8 expression, elevated the total H4K20me1 level, increased H4K20me1 bound at the mTOR promoter, upregulated mTOR signaling, and impaired autophagy. These findings suggest that Blmh is a negative regulator of mTOR signaling by controlling Hcy-related metabolite levels that influence the extent of H4K20me1 binding at the mTOR promoter [125].

**Figure 6:**
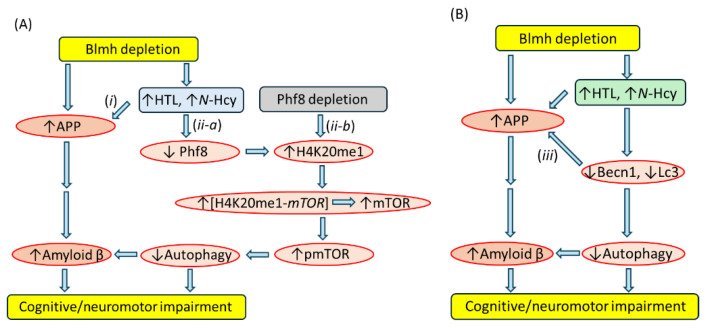
Hypothetical pathways leading to Aβ upregulation in *Blmh*^−/−^5xFAD mice. Panel (**A**) illustrates the APP (*i*) and Phf8 (*ii-a*) pathways. Panel (**B**) highlights the interaction (*iii*) between autophagy (Becn1) and APP pathways. Up and down arrows show the direction of changes. Blmh, bleomycin hydrolase; Hcy, homocysteine; HTL, Hcy-thiolactone; APP, amyloid beta precursor protein; mTOR, mammalian target of rapamycin; pmTOR, phospho-mTOR; and Phf8, plant homeodomain finger protein 8. H4K20me1-*mTOR* represents H4K20me1 bound at the *mTOR* promoter. Modified from Witucki et al. [125].

Phf8 is also a mediator directly linking Hcy-thiolactone and *N*-Hcy-protein with dysregulated mTOR signaling and its downstream outcomes such as impaired autophagy flux and upregulated Aβ accumulation, thus supplying a plausible mechanism explaining neuropathy induced by Blmh deficiency [125] (Figure 6) and explaining an association of HHcy with Alzheimer’s disease [8]. This function of Phf8 is further supported by experiments showing that *Phf8* gene silencing had the same impact on mTOR, autophagy, and Aβ as did *Blmh* gene silencing or the treatments with Hcy-thiolactone or *N*-Hcy-protein [125].

#### 5.2.5. Blmh Interacts with App, but Phf8 Does Not

Importantly, *Blmh* gene deletion upregulated App in *Blmh*^−/−^ in *Blmh*^−/−^5xFAD mice as did *Blmh* gene silencing in mouse neuroblastoma N2a-APPswe cells [125]. However, silencing the *Phf8* gene had no effect on App expression, suggesting that the Blmh interacts with App in the CNS while Phf8 does not. The Blmh–App interaction is most likely direct, as suggested by other investigators who found that human BLMH interacts with APP in vitro and that overexpressed BLMH processes human APP to Aβ in the 293-HEK and CHO cells [131]. Another report showed that rat Blmh has the ability to hydrolyze Aβ40 and Aβ42 in vitro, with fibrillar Aβ forms being more resistant than nonfibrillar Aβ [104]. Another possibility is that BLMH can regulate mTOR expression via binding to the *mTOR* promoter, supported by findings that BLMH can bind to DNA [132,133]. Further studies are needed to clarify the mechanism underlying the regulation of APP by BLMH.

Although Blmh depletion downregulated Phf8 and upregulated APP and Aβ, Phf8 depletion upregulated Aβ but not APP. These findings suggest that three pathways contribute to Aβ upregulation in a Blmh-depleted mouse brain (Figure 6) [125]. In the first pathway (*i*, Figure 6A), Hcy metabolites upregulate APP (independently of Phf8), which leads to Aβ upregulation in Blmh-depleted or Hcy-thiolactone/*N*-Hcy-protein-treated mouse brain cells. In the second pathway (*ii-a*, Figure 6A), Hcy metabolites downregulate Phf8, which upregulates mTOR signaling and thereby reduces autophagy flux resulting in Aβ upregulation due to impaired clearance. The direct depletion of Phf8 by RNA interference, independently of Hcy metabolites, also starts a similar pathway mediated by mTOR (*ii-b*, Figure 6A) that results in Aβ accumulation due to impaired autophagy. These pathways remain to be verified in future studies by testing the effects of Phf8 overexpression or mTOR downregulation (by pharmacological inhibition using rapamycin or by RNA interference) on APP and Aβ accumulation in *Blmh*-depleted cells.

#### 5.2.6. Becn1 Interacts with App

The findings that APP upregulation was accompanied by the downregulation of Becn1, a protein with a central role in autophagy initiation, in the *Blmh*^−/−^5xFAD mouse brain and in mouse neuroblastoma cells suggest that a third pathway, involving an interaction between Bcln1 and APP, contributes to Aβ upregulation [125]. In this pathway, Becn1 is a negative regulator of APP expression and processing (*iii*, Figure 6B). This conclusion is supported by prior findings showing that the level of Becn1 is significantly reduced in human AD brains compared with non-AD controls, and that the reduction in the Becn1 level in transgenic APP-overexpressing *APP*^+^*Becn*^+/−^ mice increased Aβ accumulation in neuronal cells [134]. Becn1 was also reported to regulate APP processing and turnover. The depletion of Bcln1 by siRNA in rat neuroblastoma B103/hAPPwt cells expressing human APP transgene elevated APP, Lc3, and Aβ, while the overexpression of Becn1 reduced APP level [135]. The involvement of autophagy in APP and Aβ accumulation in Blmh-depleted cells needs to be confirmed in future studies, e.g., by enhancing autophagy (e.g., with TAT-Beclin1), which should rescue APP and Aβ accumulation in these cells.

#### 5.2.7. Similar Effects of Blmh Depletion and Hcy-Thiolactone/N-Hcy-Protein on Pathways Leading to Aβ

Interestingly, *Blmh* gene deletion or HHcy induced by a high Met diet led to similar changes in the Phf8 → H4K20me1 → mTOR → autophagy pathway and Aβ accumulation [125]. These findings are consistent with the fact that the *Blmh* gene deletion and high Met diet lead to the same biochemical outcome: upregulation of Hcy-thiolactone and *N*-Hcy-protein levels [17]. Indeed, treatments of mouse neuroblastoma cells with individual metabolites that accumulate in HHcy, Hcy-thiolactone or *N*-Hcy-protein recapitulated changes in the Phf8 → H4K20me1 → mTOR → autophagy pathway and Aβ accumulation seen in Blmh-depleted or Met-supplemented HHcy wild-type mice [125]. These findings also suggest that the dysregulation of the Hcy metabolism in general would affect the Phf8 → H4K20me1 → mTOR → autophagy pathway in the CNS. Indeed, Hcy metabolites inhibit autophagy, elevate Aβ, and induce neuropathy by dysregulating the Phf8/H4K20me1-dependent epigenetic regulation of mTOR in cystathionine β-synthase-deficient mice and Cbs-silenced mouse neuroblastoma cells [30].

Blmh deficiency or HHcy induced by a high Met diet elevated the level of the methylated histone H4K20me1 via the downregulation of the histone demethylase Phf8 in the mouse brain [125]. HHcy is also known to affect DNA and protein methylation via *S*-adenosylhomocysteine (AdoHcy, an inhibitor of cellular AdoMet-dependent methylation reactions), which underlies the pathology of HHcy-associated human disease [136]. However, s possible inhibition of H4K20 histone methylase by AdoHcy would have an opposing effect; i.e., it would reduce the H4K20me1 level. The findings linking Blmh with the status of the histone H4K20me1 methylation are reminiscent of the findings showing that Pon1 deletion in mice elevated the H4K20me1 methylation level via the downregulation of Phf8 [99] (Figure 4). Thus, these two Hcy-thiolactone-detoxifying enzymes exert similar effects on H4K20me1 levels. Although there is no evidence that Blmh or Pon1 is linked to DNA methylation, these findings provide the first evidence that Blmh and Pon1 influence histone methylation.

### 5.3. Biphenyl Hydrolase-like Enzyme

The biphenyl hydrolase-like (BPHL) enzyme, also called valacyclovir hydrolase, is the third enzyme shown to use Hcy-thiolactone as a natural substrate [35,36,37]. It is a 32 kDa mitochondrial protein highly expressed in the human liver and kidney [137,138]. BPHL hydrolyzes and activates the antiviral prodrug esters valacyclovir and valganciclovir, used in the treatment of herpes simplex, herpes zoster (shingles), and herpes B [139]. However, valacyclovir was rapidly hydrolyzed to acyclovir in *Bphl*^−/−^ mice, which shows that BPHL is not obligatory for the conversion of valacyclovir to acyclovir [140]. Recent study shows that Bphl is one of the proteins associated with sperm capacitation in white boars [141].

First cloned from the breast carcinoma cells and expressed in *E. coli*, BPHL, a member of the alpha/beta hydrolase fold family, is a serine hydrolase distantly related to other members of the serine hydrolase family [137,138]. The *BPHL* gene is located on human chromosome 6p25 in a locus with other serine hydroxylases.

Crystallographic studies showed that human BPHL has the catalytic triad S122-D227-H255, a serine hydrolase consensus sequence GSXSG, and a unique binding mode and the specificity for esters of α-amino acids [142]. The α-amino acid specificity, including the ability of BPHL to hydrolyze L-Met methyl ester shared with the Hcy-thiolactone-hydrolyzing enzyme BLMH [34], suggested that BPHL could also hydrolyze Hcy-thiolactone. Indeed, this prediction, was substantiated by conference reports published in 2010–2011 [35,36] and a report published in a 2014 Plos One article [37].

BPHL, BLMH, and PON1 differ in their specificities towards non-physiological substrates for which they have been originally named and in catalytic efficiencies towards Hcy-thiolactone (Table 1). The catalytic efficiency of BPHL in the Hcy-thiolactone hydrolytic reaction is higher than that of BLMH or PON1, suggesting that BPHL can have a significant contribution to Hcy-thiolactone detoxification in vivo [37].

#### Consequences of Bphl Ablation

A recent study found that the *BPHL* gene is overexpressed in lung cancer and promotes lung carcinogenesis and that the downregulation of the BPHL expression by RNA interference inhibited tumor growth and metastasis by impairing the progression of the cell cycle and inducing apoptosis in A549, NCI-H1975, and NCI-H-1299 human lung carcinoma cell lines [143]. The deletion of the *Bphl* gene in mice decreased circulating creatinine levels in males, suggesting a kidney function defect (http://www.informatics.jax.org/allele/allgenoviews/MGI:5548556 (accessed on 15 July 2024)).

A recent report has shown that the deletion of the *Bphl* gene in mice significantly attenuated Hcy-thiolactone turnover in vivo [144], similar to the impairment of Hcy-thiolactone turnover in *Blmh*^−/−^ mice [48]. Notably, silencing the *Bphl* gene by RNA interference in mouse neuroblastoma N2a-APPswe cells caused changes in the Phf8 → H4K20me1 → mTOR → autophagy pathway and APP/Aβ levels characteristic of AD [144], similar to the changes seen in mouse neuroblastoma N2a-APPswe cells in which *Pon1* [99] or *Blmh* [125] was silenced by RNA interference.

## 6. Conclusions and Future Direction

Hcy-thiolactone, a product of an error-correcting reaction during protein biosynthesis, is generated in the human body when Hcy is selected in place of methionine by methionyl-tRNA synthetase. Hcy-thiolactone is a chemically reactive thioester metabolite that modifies protein lysine residues in a process called *N*-homocysteinylation. The modification causes protein damage/aggregation, a hallmark of many diseases, including Alzheimer’s. Hcy-thiolactone-detoxifying enzymes—serum paraoxonase PON1 carried in the circulation on high-density lipoprotein, cytoplasmic bleomycin hydrolase BLMH, and mitochondrial biphenyl hydrolase-like enzyme BPLH—protect the human body proteins from Hcy-thiolactone/*N*-homocysteinylation-associated damage. The depletion of any of these enzymes elevates Hcy-thiolactone and *N*-Hcy-protein, which dysregulate the Phf8 → H4K20me1 → mTOR → autophagy pathway and upregulate APP, causing Aβ accumulation, a hallmark of Alzheimer’s disease.

The epigenetic consequences and mechanisms of dysregulated histone methylation, mTOR signaling, and autophagy caused by Hcy and related metabolites, and their roles in Hcy-related diseases, including AD, will provide a fertile field for future systematic studies that are needed to elucidate the roles of Hcy-thiolactone-detoxifying enzymes— PON1, BLMH, and BPHL—in human health and disease. Future studies are also needed to elucidate how metabolic signals from Hcy and related metabolites are transmitted into cells and affect biochemical/physiological outcomes. Recent findings show that the effects of Hcy, Hcy-thiolactone, and *N*-Hcy-protein on PHF8, mTOR, and autophagy are mediated by microRNAs [145]. Whether microRNAs affected by Hcy metabolites are involved in AD pathology remains to be studied. Such studies could reveal means to control Hcy-thiolactone levels and to ameliorate protein damage, thus contributing to the development of new strategies for AD prevention and treatment.

## Figures and Tables

**Table 1:** Substrate specificities of human Hcy-thiolactone hydrolyzing enzymes *.

Substrate	PON1 (%)	BLMH (%)	BPHL (%)
*L*-Hcy-thiolactone (k_cat_/K_m_)	100 (10 M^−1^s^−1^)	100 (10^3^ M^−1^s^−1^)	100 (7.7 × 10^4^ M^−1^s^−1^)
*D*-Hcy-thiolactone	24	<1	ND
γ-Thiobutyrolactone	545	<1	<0.001
*N*-Acetyl-*D*,*L*-HTL	<1	<1	<0.001
*L*-Hse-lactone	++++	−	+++
*L*-Met methyl ester	<1	++	30
*L*-Cys methyl ester	<1	++	++
*L*-Lys methyl ester	ND	−	−
*L*-Phe ethyl ester	0	ND	16
*Nε*-Hcy-aminocaproate	ND	++++	ND
Val(*Nε*-Hcy-Lys)	ND	++++	ND
HcyLeuAla	ND	++++	ND
Bleomycin	ND	500	ND
Paraoxon	330	−	ND
Phenyl acetate	280,000	−	<0.001
Valacyclovir	–	ND	22

* Data for PON1 and BLMH are from refs. [33,34], respectively, and for BPHL from refs. [35,36,37]. Multiple ‘+’ symbols indicate relative enzymatic activity for indicated substrates; a ‘−’ symbol means no activity. Hcy, homocysteine; Hse, homoserine; and ND, not determined.

**Table 2:** Hcy-thiolactone-hydrolyzing activity is absent in serum from *Pon1*^−/−^ mice.

Genotype	Hcy-Thiolactone Hydrolase Activity (%)		Paraoxonase Activity (%)	
	Male	Female	Male	Female
*Pon1* ^−/−^	0	0	0	0
*Pon1* ^+/^^−^	51	30	50	40
*Pon1* ^+/+^	100	73	100	70

Data from Jakubowski [33] and Borowczyk et al. [49].

## Data Availability

The data that support the findings of this study are available in the methods of this article.
